# Computational discovery and RT-PCR validation of novel *Burkholderia *conserved and *Burkholderia pseudomallei *unique sRNAs

**DOI:** 10.1186/1471-2164-13-S7-S13

**Published:** 2012-12-07

**Authors:** Jia-Shiun Khoo, Shiao-Fei Chai, Rahmah Mohamed, Sheila Nathan, Mohd Firdaus-Raih

**Affiliations:** 1 School of Biosciences and Biotechnology, Faculty of Science and Technology, Universiti Kebangsaan Malaysia, 43600 UKM Bangi, Malaysia; 2 Codon Genomics SB, Jalan Bandar 18, Pusat Bandar Puchong, Selangor Darul Ehsan, Malaysia; 3 Malaysia Genome Institute, Jalan Bangi Lama, 43000 Kajang, Malaysia

## Abstract

**Background:**

The sRNAs of bacterial pathogens are known to be involved in various cellular roles including environmental adaptation as well as regulation of virulence and pathogenicity. It is expected that sRNAs may also have similar functions for *Burkholderia pseudomallei*, a soil bacterium that can adapt to diverse environmental conditions, which causes the disease melioidosis and is also able to infect a wide variety of hosts.

**Results:**

By integrating several proven sRNA prediction programs into a computational pipeline, available *Burkholderia *spp. genomes were screened to identify sRNA gene candidates. Orthologous sRNA candidates were then identified via comparative analysis. From the total prediction, 21 candidates were found to have Rfam homologs. RT-PCR and sequencing of candidate sRNA genes of unknown functions revealed six putative sRNAs which were highly conserved in *Burkholderia *spp. and two that were unique to *B. pseudomallei *present in a normal culture conditions transcriptome. The validated sRNAs include potential cis-acting elements associated with the modulation of methionine metabolism and one *B. pseudomallei*-specific sRNA that is expected to bind to the Hfq protein.

**Conclusions:**

The use of the pipeline developed in this study and subsequent comparative analysis have successfully aided in the discovery and shortlisting of sRNA gene candidates for validation. This integrated approach identified 29 *B. pseudomallei *sRNA genes - of which 21 have Rfam homologs and 8 are novel.

## Introduction

Small RNAs (sRNAs) are known to function as regulatory or catalytic molecules in bacteria with sequences normally ranging from ~50-250 nt in length and located in the intergenic regions (IGRs) [1,2]. Although sRNAs with catalytic functions have been reported [3,4], many of these molecules are known or believed to function as regulatory nucleic acid elements that target near, or at, the translation start site of their dedicated mRNA targets via imperfect sequence complementarity [5-7]. In *E. coli*, less than 100 sRNAs, accounting for ~0.3% of the genome, have been reported [8-10]. Although these riboregulators represent only a small fraction of the prokaryotic genome, they have been shown to play essential regulatory roles in bacteria, including cell surface modulation [11], plasmid number control [12], stress adaptation [13], quorum sensing [14] and carbon storage [15]. Other regulatory sRNAs interact with and modulate cellular protein activities [16].

In pathogenic bacteria, sRNAs have been associated with regulatory networks that modulate the adherence to, and invasion into the host cell [17,18], environmental adaptation [19,20] as well as virulence and pathogenicity [17,18,20-23]. In several bacterial pathogens, including *Salmonella typhimurium *[24], *Vibrio cholerae *[25], *Yersinia enterocolitica *[26], *Brucella abortus *[23] and *Pseudomonas aeruginosa *[27], deletion of the *hfq *gene which encodes the RNA chaperone Hfq, has been shown to severely attenuate virulence. The Hfq protein is known to facilitate the pairing interaction between sRNAs and their target mRNAs [28]. Identification and analysis of sRNAs in pathogenic bacteria may improve current understanding on the molecular mechanisms of host adaptation and virulence. Hence, we carried out a computational based analysis of available *Burkholderia *spp. genomes to identify potential sRNA sequences and to further delineate sRNAs that are present only in the pathogenic members.

Members of the *Burkholderia *genus also play important roles as environmental saprophytes. One species of this genus, *B. pseudomallei*, is the causative agent of melioidosis, a disease endemic to Southeast Asia and northern Australia. This species reportedly has a highly dynamic genome and versatile phenotypes [29-31], thus contributing to its capability to infect nearly all cell types, resulting in a wide spectrum of disease symptoms that confounds diagnosis and delays prompt treatment. *B. pseudomallei *is an effective pathogen of a broad range of hosts (amoeba [32], nematodes [33], dolphins [34], birds, camels, alpacas, sheep [35], humans and even plants [36]). The enigma of *B. pseudomallei *is further compounded in having an extremely prolonged latent infection capacity [36] and has been shown to be capable of surviving in a nutrient-free environment for 16 years [37].

*B. pseudomallei *is believed to have an array of virulence and pathogenicity factors, including a toxin which is a deamidase named *Burkholderia *Lethal Factor 1 (BLF1) that targets the translation initiation factor eIF4a [38]. However, the regulation and delivery mechanism of BLF1 to the target protein remains unclear. To date, the mechanisms of adaptation to environmental stress and changes have not been conclusively identified, however a large number of sRNA genes have been reported for *B. cenocepacia *J2315, another pathogenic member of the *Burkholderia *genus [39]. These sRNAs were proposed to be responsible for the bacterium's complexity, phenotypic variability and ability to survive in a remarkably wide range of environments [39].

At present, one can opt for either a knowledge-based approach or a *de novo *approach for sRNA discovery in a bacterial genome. Knowledge-based techniques search for homologues of known sRNAs based on specific features of the sequences and will usually include upstream regulatory elements, sequence and structural characteristics and downstream targets as a search profile. A number of knowledge-based programs were developed to identify particular sets of sRNAs through homology analysis. One such program, Infernal [40], was the workhorse used to build the Rfam database [9]. However, predictions relying on homology information limit the applications of such programs to sRNA genes with known homologues and therefore, the methods are insufficient in situations where many if not most bacterial sRNAs remain unidentified. A *de novo *approach can serve a complementary role in predicting novel sRNA genes that are beyond the profile scope of knowledge-based approaches. The basis of a *de novo *search lies in the common features of sRNAs in the genomes - sequence and structural conservation, shared physical co-localization, structural stability, existence of transcriptional signals and GC bias - without prior knowledge of the sRNAs to be discovered. Such an approach was applied with various sRNA gene finders such as QRNA [41], RNAz [42,43], sRNAPredict [44,45] and sRNAscanner [46]. In this paper, we report the development of a computational pipeline that integrated successful sRNA prediction programs to identify candidate sRNA genes in *B. pseudomallei *and subsequent validation by RT-PCR and Sanger sequencing.

## Methods

### Development of the sRNA gene detection pipeline

**Figure 1:**
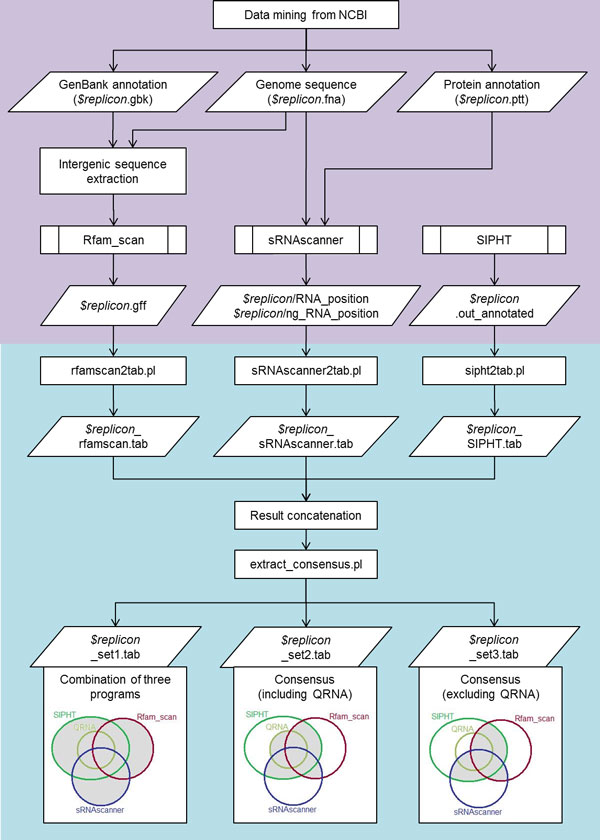
**Pipeline for bacterial sRNA gene prediction**. The steps in the purple zone (top) were executed manually while the steps in the blue zone (bottom) were automated using Perl script GetRNA.pl developed in this study. The variable *$replicon *refers to the replicon tested.

A computational pipeline for bacterial sRNA gene prediction was developed by integrating the output of three published sRNA detection programs; Rfam_scan [9], SIPHT [48] and sRNAscanner [46]. The pipeline consists of a number of core programs for data format conversion and consensus identification and a main program (GetsRNA.pl) that controls the data flow between the elementary scripts (Figure 1). sRNA candidates were then named according to the following convention Bp[Chromosome number]_[candidate number for program]_[program name] eg. *Bp1_Cand612_SIPHT*.

The intergenic sequences (here, defined as sequences between annotated ORFs) of the replicons were extracted using Artemis v12.0.3 [49] and searched against the Rfam database v10.0 by executing the script rfam_scan.pl v1.0. The supporting software used for the search included BLAST v2.2.22 [50], Infernal v1.0, Perl v5.10.0 and BioPerl v1.6.0.

SIPHT searches were restricted to detect sRNA genes within the range of 30-550 nucleotides and executed via the web server (URL: http://newbio.cs.wisc.edu/sRNA/). Other parameters were optimized as suggested [48]; i.e., maximum E value: 1e-15, minimum TransTerm confidence value: 87, maximum FindTerm score: -10, maximum RNAMotif score: -9. All replicons, except the replicon of interest, were included as a partner replicon for the search.

The program sRNAscanner_Ubuntu10 (released 31 August 2010) was used to screen both the forward and reverse strands of the query replicon. The searches were restricted to intergenic regions and the sRNA length for prediction was set to 30-550 nucleotides. All other parameters were left at their default values, i.e. 3 provided input matrices: 35box_sRNA.matrix (cut-off: 2), 10box_sRNA.matrix (cut-off: 2), terminator.txt.matrix (cut-off: 3); spacer range between [-35] & [-10] promoter boxes: 12-18; unique hit value: 200; minimum cumulative sum of score (CSS): 14.

### Genome sequences, annotation files and databases

The genome sequences of 11 *Burkholderia *spp and 3 *Ralstonia *spp (.fna extensions), annotation files (.gbk and .ptt extensions) and the complete genomic sequences of RefSeq-release47 (.genomic.fna extensions) were obtained from NCBI (Additional file 1). The genome sequences of five local strains of *B. pseudomallei *(unpublished data) were used for cross-referencing purposes. The Rfam database v10.0, both .fasta and .cm extensions for 1,446 sRNA families, was downloaded from ftp://ftp.sanger.ac.uk/pub/databases/Rfam/.

### Comparative analysis

The intergenic sequences of *B. pseudomallei *K96243 were compared to sRNA candidates predicted in the *Ralstonia *and *Burkholderia *genomes using blastn v2.2.21 (parameters: -e 1e-5 -r 1 -q -1 -G 1 -E 2 -W 9 -F "m D"). The results were visualized using ACT v9.0.3 [51] and the gene physical co-localization for the sRNAs of interest were investigated.

### Secondary structure prediction

The secondary structures of the sRNA transcripts were predicted using mfold (unafold v3.8) [52] and RNAfold (ViennaRNA v1.8.4) [53]. The default parameters or standard conditions for RNA folding were accepted (37°C, 1M NaCl, no divalent ions). The predicted structures were visualized using VARNA v3.7 [54].

### Homologue detection

Sequences for sRNAs of interest were globally aligned and consensus secondary structures were predicted using LocARNA [55] via its web service (URL: http://rna.tbi.univie.ac.at/cgi-bin/LocARNA.cgi). The default parameters for scoring the alignments were accepted (RIBOSUM85_60 matrix, Indel-opening score: -500, Indel score: -350, structure weight: 180, avoid lonely base-pairs). Covariance models representing the alignments with consensus structures were built, calibrated and searched against complete genome sequences in the RefSeq database release 47 using Infernal v1.0 with an E-value ≥ 1e-3.

### *B. pseudomallei *strain and RNA extraction

The *B. pseudomallei *D286 human isolate was obtained from the Pathogen Laboratory, School of Biosciences and Biotechnology, Faculty of Science and Technology, Universiti Kebangsaan Malaysia, Malaysia. Stock cultures were stored at -70°C and routinely cultured on brain-heart infusion agar (BHIA) (Pronadisa Hispanlab, South Africa) at 37°C [56]. Bacteria from a stock culture were taken and streaked on Ashdown agar, and incubated at 37°C for 48 hours. A single colony was picked from the plate and inoculated into Brain Heart Infusion broth (BHIB) overnight. The following day, the culture was diluted 1:100 and grown in BHIB until the OD_600 _reached 0.6 - 1.0. Total RNA was extracted using TRIzol^® ^LS Reagent (Invitrogen, Carlsbad, CA) and purified using Ambion's DNA*free*™ DNase Treatment and Removal Reagents (Life Technologies, Carlsbad, CA).

### Reverse transcription polymerase chain reaction (RT-PCR) and Sanger sequencing

The purified RNA was reverse transcribed into cDNA with an oligo(dT)18 primer using RevertAid First Strand cDNA Synthesis Kit (Fermentas, Hamburg, Germany). The cDNA produced was used as the template for PCR together with primers that were designed based on the sequences of sRNA candidates (Additional file 2). Amplification reactions were performed in a total volume of 25 μL consisting 10x PCR buffer, 10 mmol/L of dNTP mix, approximately 100 ng of cDNA, 25 pmol of each primer, 1.0 U Taq polymerase (Promega, Madison, WI) and distilled water. Mastercycler^® ^personal (Eppendorf, Hamburg, Germany) was used to perform gradient PCR, with an initial denaturation step of 2 minutes at 95°C, followed by 35 amplification cycles of 30 seconds at 95°C, 30 seconds at 54-62°C, and 30 seconds at 72°C, and a final extension of 2 minutes at 72°C. Amplified products were analyzed by 3% agarose gel electrophoresis with O'GeneRuler™ Low Range DNA Ladder (Fermentas, Vilnius, Lithuania) run in parallel. PCR products were purified with the QIAquick Gel Purification Kit (Qiagen, Germany) and used in the reaction with the BigDye^® ^Terminator v3.1 Cycle Sequencing Kit (Applied Biosystem, Foster City, CA). Three biological replicates were carried out for each RT-PCR primer sets. The PCR products were then sequenced on the ABI Prism^® ^3100 AVANT DNA Sequencer. The sequences obtained were analyzed using BioEdit v7.3.1.0 and compared with the genome sequence of *B. pseudomallei *D286 human isolate.

## Results and discussion

### Pipeline development and performance assessment

Several computational approaches for sRNA discovery have been used on various bacterial genomes to successfully identify and validate tens to hundreds of putative sRNA genes (Table 1). Due to resource limitations, it is common practice for only a limited number of the hundreds to thousands of computationally identified sRNAs to be selected for experimental verification and characterization. The percentage of validated sRNAs relative to the total number of predicted candidates can be as high as 37.6% as in the case for *Streptomyces coelicolor *[47] to as low as 0.1% in *B. cenocepacia *[39]. The percentage of verified sRNAs over the number of computationally predicted candidates which were tested is similarly varied; 78.7% for *S. coelicolor *and 1.88% for *B. cenocepacia*. In some cases, the verification experiments are inconclusive due to the uncertainty of whether a target transcript was transcribed under the particular experimental or culture conditions used. As a result, the number of sRNAs validated experimentally is usually smaller than the number of sRNAs originally selected for verification from the computationally predicted list (Table 1).

**Table 1:** Discovery and verification of bacterial sRNAs in previous studies.

Bacteria	Computational discovery method	Verification method	Number of sRNAs			Reference
		
			Predicted	Tested	Verified	
*Escherichia coli*	QRNA	Northern blot	275	49	11	[64]
*Escherichia coli*	*Pftools2.2 *& RNAMotif	Northern blot	227	8	7	[65]
*Burkholderia cenocepacia*	QRNA	Microarray	3,441	213	4	[39]
*Streptomyces coelicolor*	BLAST & TransTermHP	RT-PCR & microarray	37	32	20	[66]
*Synechocystis *PCC6803	RNAz	Northern blot	383	2	2	[67]
*Staphylococcus aureus*	RNAsim	Northern blot	774	36	11	[68]
*Escherichia coli*	Anonymous program	Northern blot	601	6	3	[69]
*Salmonella enterica *Typhimurium	sRNAscanner	Northern blot	156	16	6	[46]
*Streptomyces coelicolor*	RNAz & nocoRNAc	Microarray	843	403	317	[47]
*Burkholderia pseudomallei*	SIPHT, sRNAscanner & Rfam_scan	RT-PCR	1306	15	8	This study.

**Figure 2:**
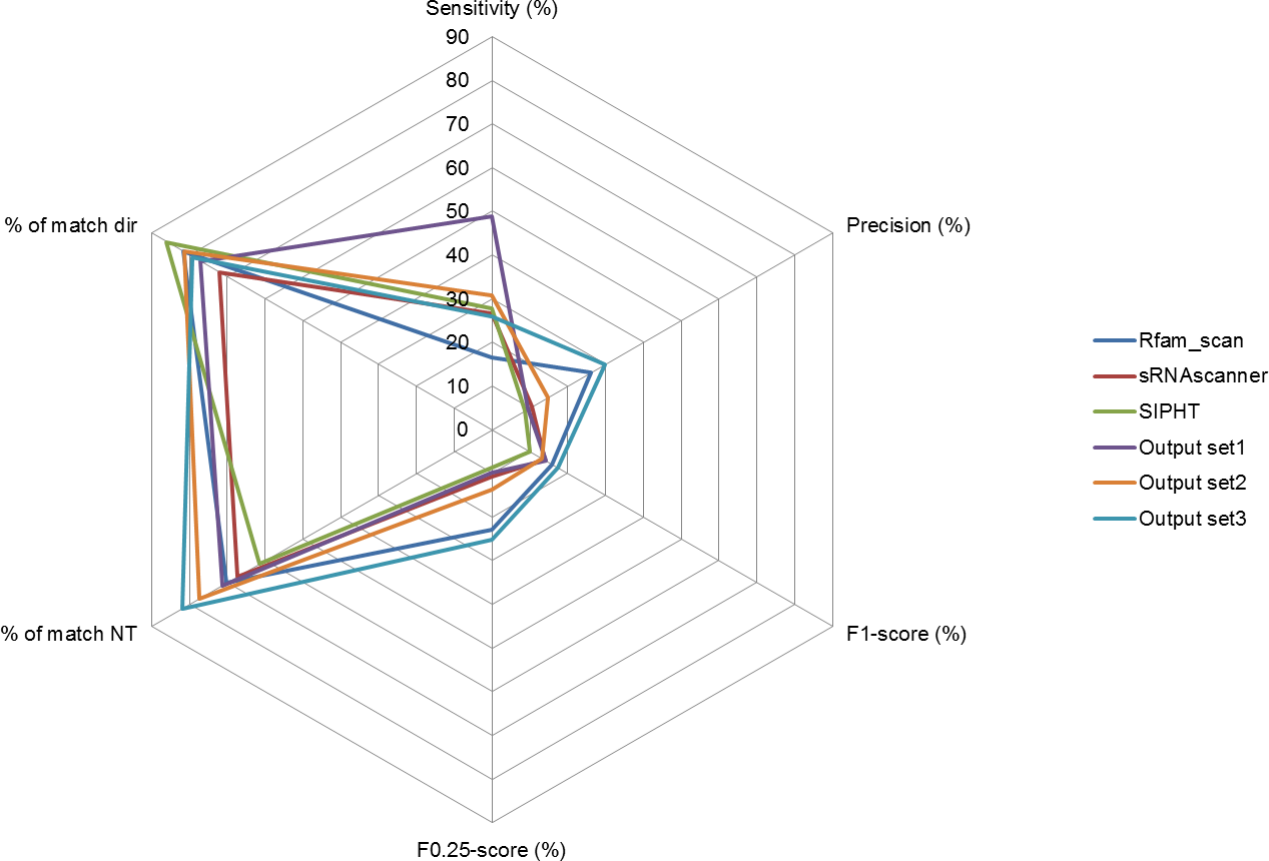
**Quantitative assessment of the performance for the different sRNA prediction methods on benchmark datasets**.

Three different sRNA predictors Rfam_scan [9], SIPHT [48] and sRNAscanner [46], each with different sets of criteria used to identify bacterial sRNAs, were selected to be the elementary programs in the sRNA prediction pipeline developed. The integrated sRNA prediction pipeline (Figure 1) enabled the discovery of sRNA genes in the target genome sequences of organisms from the order Burkholderiales via analysis of the consensus results. The performance of the pipeline was initially assessed using the benchmark datasets and assessment method reported by Lu et al. [57]. The three sets of outputs from the pipeline, as well as predictions by individual elementary programs in the pipeline were evaluated using the ten sets of benchmark sRNAs (Figure 2). The highest mean sensitivity was achieved by compiling all the predictions (output set1) resulting in a retrieval rate of 48.88%; the next most sensitive approach, with 30.65% retrieval, was output set2, i.e. an overlap of the different outputs integrated by our pipeline to derive a consensus predicted sRNA list (which included QRNA output). In the case of precision assessment, output set3, i.e. consensus predicted sRNA list (excluding QRNA output) outperformed other methods by achieving a mean precision of 29.90%. By using the F_1 _measure (which puts equal weight on sensitivity and precision) and F_0.25 _measure (which puts the weightage on precision at four times more than sensitivity) as proxies of prediction accuracy [58]; it was revealed that output set3 from the pipeline achieved the highest mean performance, i.e., 17.35% and 25.26% respectively for each measure. In addition, output set3 also outperformed the other prediction methods in predicting the extent of sRNAs by identifying 81.87% of sRNA nucleotides on average. However, the highest ability to correctly identify the transcription directions of these sRNAs was achieved by SIPHT, i.e., 86% of the time on average. The pipeline enabled us to carry out the computational annotation of sRNA genes for available *Burkholderia *genomes by analyzing the conservation of predicted candidates in different species from the genuses *Burkholderia *and *Ralstonia*. Additionally, by interrogating the genome sequences of different *B. pseudomallei *isolates, we were able to identify conserved sRNA candidates that are unique to *B. pseudomallei *and are discussed further below.

### sRNA searches in *B. pseudomallei *and other related species

**Figure 3:**
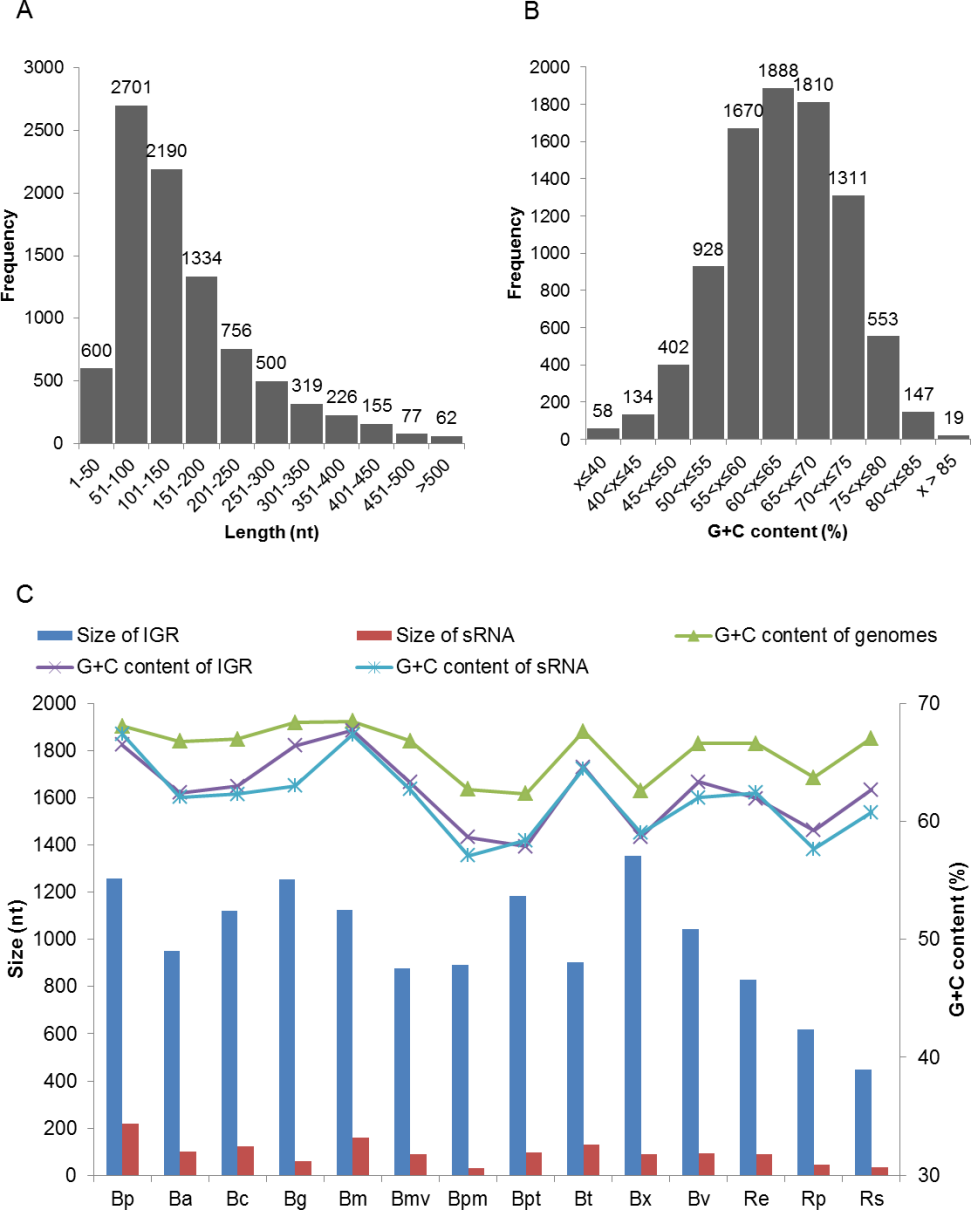
**Overview of sRNA candidates**. (A) Length distribution. (B) G+C content distribution. (C) Properties of genomes, IGRs and sRNA candidates by species: Column graph - Total IGR and sRNA candidate sizes. Line graph - Overall G+C content of genome, IGR and sRNA candidate sequences. Bp - *B. pseudomallei*, Ba - *B. ambifaria*, Bc - *B. cenocepacia*, Bg *- B. glumae*, Bm - *B. mallei*, Bmv - *B. multivorans*, Bpm - *B. phymatum*, Bpt - *B. phytofirmans*, Bt *- B. thailandensis*, Bx - *B. xenovorans*, Bv - *B. vietnamiensis*, Re - *R. eutropha*, Rp - *R. pickettii*, Rs - *R. solanacearum *(further details of the species analysed are available in Additional file 1).

Fourteen genomes (Additional file 1) including *B. pseudomallei *were searched for sRNA genes using the pipeline developed. A total of 8,920 individual sRNA candidates were returned from the searches (output set1), with the smallest number, 193, for *R. solanacearum*, and the largest number, 1,306, for *B. pseudomallei*. Files containing the locations of all predicted sRNA genes are available as Additional file 3. The 8,920 sRNA candidates identified, varied in length between 24 and 551 nt (Figure 3A). The majority of the sRNA candidates (78.26%) were 51-250 nt in length. The G+C percentage of the sRNA candidates ranged from 25.44% to 89.15%. The G+C content distribution of sRNA candidates (Figure 3B) suggested that most of the sRNA candidates (82.72%) have a G+C content higher than 55%. As sRNAs are diverse in both functions and mechanisms of action, various G+C content in the sRNAs would be expected to fulfill different requirements of stability. From previous studies [8,59], the G+C content of sRNAs were found to be higher than in the associated IGRs. However, we found that the overall G+C content of sRNAs predicted for the 14 genomes in our work was generally comparable to the G+C content of the IGRs (Figure 3C, line graph). This could however be attributed to the fact that the bacterial genomes analyzed are already of high G+C percentage, i.e. 62.35% to 68.49%. Additionally, false positive sRNA candidates obtained during the predictions could also be one of the factors contributing to this observation. The overall size of the sRNA candidates retrieved was not affected by the overall size of the IGRs from where they are predicted (Figure 3C, column graph).

### Comparative analysis

The IGR sequences identified for *B. pseudomallei *were compared against the 8,920 sRNA candidates using a BLAST-based (blastn) method. The purpose for this comparative analysis is to determine the conservation of sRNA candidates among the closely related bacterial species. As mis-annotations occur in genomes and each of the gene predictors have their own limitations, it was therefore no surprise to detect putative sRNAs from this comparison but not predicted by the sRNA search pipeline. A total of 1,213 out of 4,978 (approximately 24%) *B. pseudomallei *IGRs were predicted to contain at least one sRNA gene. The complete results list for this comparative analysis is provided as Additional file 4. As two or more sRNA genes could be predicted at the same strand and location, the overlapping candidates were merged before further analysis. For example, if gene A (location: 100 - 200) overlaps with gene B (location 150 - 250), the genes were merged into gene C (location: 100 - 250).

The comparative analysis computationally identified 21 sRNAs in *B. pseudomallei *that are homologous to previously reported sRNAs (Table 2). These sRNAs include 13 cis-regulatory elements, 6 trans-acting RNAs, 1 ribozyme and 1 sRNA with unknown function. Comparison of the sRNA sequences and predicted secondary structures with entries in Rfam computationally verified the sRNAs predicted (Additional file 5). We cross-referenced the predicted sRNAs with Rfam and found that several of the predicted sRNAs were not noted in the database, while one sRNA recorded in Rfam was missed by our pipeline although the rest were correctly designated (Table 2). The sRNA gene missed during the prediction, LR-PK1 (location: 2314148 - 2314399, reverse strand of chromosome 1), was found to overlap with the *infC *gene (location: 2314163 - 2314699) located on the same strand of the same chromosome. The gene was not located in the IGRs and therefore it was not predicted in the sRNA gene detection pipeline, which was designed to search for sRNA genes only in the IGRs.

**Table 2:** List of *B. pseudomallei *sRNA sequences with their corresponding sRNA families as reported in Rfam.

**No**.	sRNA	Chr/strand	Coordinates	Coordinates from Rfam	Type
1.	Cobalamin.1	1/+	1133342..1133609	(no record)	Riboswitch
2.	Cobalamin.2	1/+	2072691..2072897	2072691..2072897	Riboswitch
3.	Cobalamin.3	1/-	2090548..2090794	(no record)	Riboswitch
4.	Cobalamin.4	1/+	2090844..2091164	(no record)	Riboswitch
5.	TPP.1	1/-	1504117..1504234	1504117..1504234	Riboswitch
6.	TPP.2	1/-	3753305..3753517	3753386..3753517	Riboswitch
7.	TPP.3	2/-	1490479..1490584	1490479..1490584	Riboswitch
8.	FMN	1/+	772307..772458	772307..772458	Riboswitch
9.	Glycine	1/+	3984000..3984174	3984000..3984108	Riboswitch
10.	SAH_riboswitch	1/-	3907800..3907867	3907800..3907867	Riboswitch
11.	Mini-ykkC	1/+	1359254..1359300	1359254..1359300	Putative riboswitch
12.	sucA	1/-	2274625..2274707	2274625..2274707	Putative riboswitch
13.	yybP-ykoY	1/+	3066135..3066276	(no record)	Putative riboswitch
14.	LR-PK1*	1/-	(not predicted)	2314148..2314399	Cis-acting RNA
15.	isrK	1/-	98332..98411	(no record)	Hfq-binding RNA
16.	6S	1/-	1132173..1132396	1132214..1132395	Trans-acting RNA
17.	SRP_bact	1/+	1735400..1735501	1735400..1735501	Trans-acting RNA
18.	tmRNA	1/+	3041943..3042311	3041943..3042311	Trans-acting RNA
19.	Anti-hemB	1/-	3790883..3790964	(no record)	Trans-acting RNA
20.	CRISPR-DR28	1/+	3578911..3578934	(no record)	Trans-acting RNA
21.	RNaseP_bact_a	1/-	3481314..3481770	3481359..3481770	Ribozyme
22.	P9	2/-	1749221..1749373	1749223..1749307	Gene

* The LR-PK1 sRNA was not predicted in this study but has been identified in Rfam.

Excluding the 21 homologues to known sRNAs, 20 previously undescribed candidates (also referred to in this paper as novel sRNAs) that were conserved in at least eight out of the fourteen bacterial genomes analyzed were selected for predicted secondary structure comparison where the calculated secondary structures were visually examined. A total of twelve sRNAs with perceivably conserved secondary structures were selected for experimental validation (discussed in the next section).

In order to verify the conservation of the twelve sRNA candidates above within the bacterial kingdom, we generated covariance models for these sRNAs using the sequences from *B. pseudomallei, B. mallei, B. thailandensis, B. cenocepacia *and *R. solanacearum *and searched against the complete genomic sequences in the RefSeq database using the Infernal program [40] (full results available in Additional file 6). Genes that are unique to *B. pseudomallei *are of interest because they may help explain the unique features that are not found in other relatives (even the very close ones), in addition to being potential biomarkers for melioidosis. We initially sorted a list of 193 sRNA candidates from *B. pseudomallei *with no homologues detected during the comparative analysis and searched the 13 genomes of close relatives (all *Burkholderia spp*. and *Ralstonia spp*. except *B. pseudomallei*) by using blastn for similar sequences. The genes with no similar sequences detected were screened again against nine other strains of *B. pseudomallei *(Additional file 1) to confirm their occurrence in all (or most) of the *B. pseudomallei *strains. Three sRNA candidates were identified from the screening as novel sRNAs unique to *B. pseudomallei *and their transcription under normal growth conditions were tested using RT-PCR (Table 3).

**Table 3:** List of RT-PCR validated sRNA genes in conserved in *Burkholderia *and unique to *Burkholderia pseudomallei*.

	Name	Chr/Strand	Start - end/Length	GC content	Conservation (Infernal search)
***Highly conserved in Burkholderia***					
	Bp1_Cand449_SIPHT*	1/-	110185 - 110354/170	50.59%	Bacteria (detected in Proteobacteria, Bacteroidetes, Firmicutes, etc)
	Bp1_Cand612_SIPHT	1/-	2290411 - 2290508/98	52.04%	*Burkholderia*
	Bp1_Cand684_SIPHT	1/-	2768674 - 2768787/114	64.04%	Bacteria (detected in Actinobacteria, Cyanobacteria, Firmicutes, etc)
	Bp1_Cand697_SIPHT	1/-	2887980 - 2888055/76	64.47%	*Burkholderia*
	Bp1_Cand738_SIPHT	1/-	3154052 - 3154260/209	50.72%	*Burkholderia*
	Bp1_Cand871_SIPHT^	1/+	4031759 - 4031986/228	59.21%	*Burkholderia*
	Bp2_Cand287_SIPHT	2/-	2326038 - 2326224/187	62.57%	Proteobacteria (predominantly in Burkholderiales, detected in Deltaproteobacteria and Gammaproteobacteria)
***Unique to B. pseudomallei***					
	Bp2_Cand11_sRNAscanner	2/-	892370 - 892562/193	36.27%	*B. pseudomallei*
	Bp2_Cand77_SIPHT	2/+	575285 - 575425/141	57.45%	*B. pseudomallei*

* Bp1_Cand449_SIPHT and Bp1_Cand846_SIPHT were verified using the same pair of primers with only Bp1_Cand449_SIPHT also confirmed via sequencing.

^ Bp1_Cand871_SIPHT was not confirmed via sequencing due to ambiguous sequencing results.

### Validation of novel sRNAs using RT-PCR

**Figure 4:**
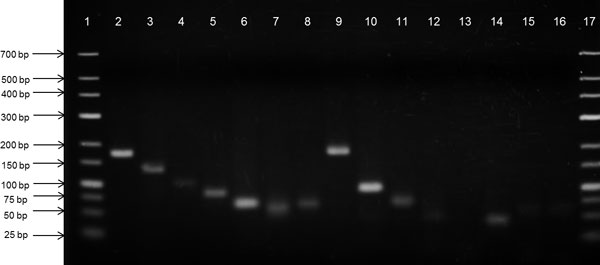
**RT-PCR validation of fifteen sRNA candidates**. Electrophoresis of PCR amplicons of 15 novel sRNAs on 3% agarose gel. Lane 1 & 17: O'GeneRuler™ Low Range DNA Ladder (Fermentas, Vilnius, Lithuania), Lane 2-16: 15 sRNA candidates and positive control (Bp2_Cand287_SIPHT, Bp1_Cand449_SIPHT/Bp1_Cand846_SIPHT, Bp2_Cand11_sRNAscanner, Bp1_Cand612_SIPHT, Bp2_Cand77_SIPHT, Bp1_Cand684_SIPHT, Bp1_Cand697_SIPHT, Bp1_Cand738_SIPHT, Bp1_Cand871_SIPHT, positive control, Bp1_Cand506_SIPHT, Bp1_Cand507_SIPHT, Bp2_Cand393_SIPHT, Bp1_Cand620_SIPHT, Bp1_Cand732_SIPHT).

A total of fifteen sRNA candidates were selected from the comparative analysis for further verification by RT-PCR of the total RNA extracted from *B. pseudomallei *D286. Eight candidates were detected in the RT-PCR experiment, each producing PCR products of the expected size with sequences that matched the predictions (Figure 4; Additional file 2). Two candidates, Bp1_Cand449_SIPHT and Bp1_Cand846_SIPHT were tested using the same pair of primers, however subsequent sequencing of the amplified products confirmed only the sequence for Bp1_Cand449_SIPHT.

### Analysis of novel sRNAs in *Burkholderia pseudomallei*

**Figure 5:**
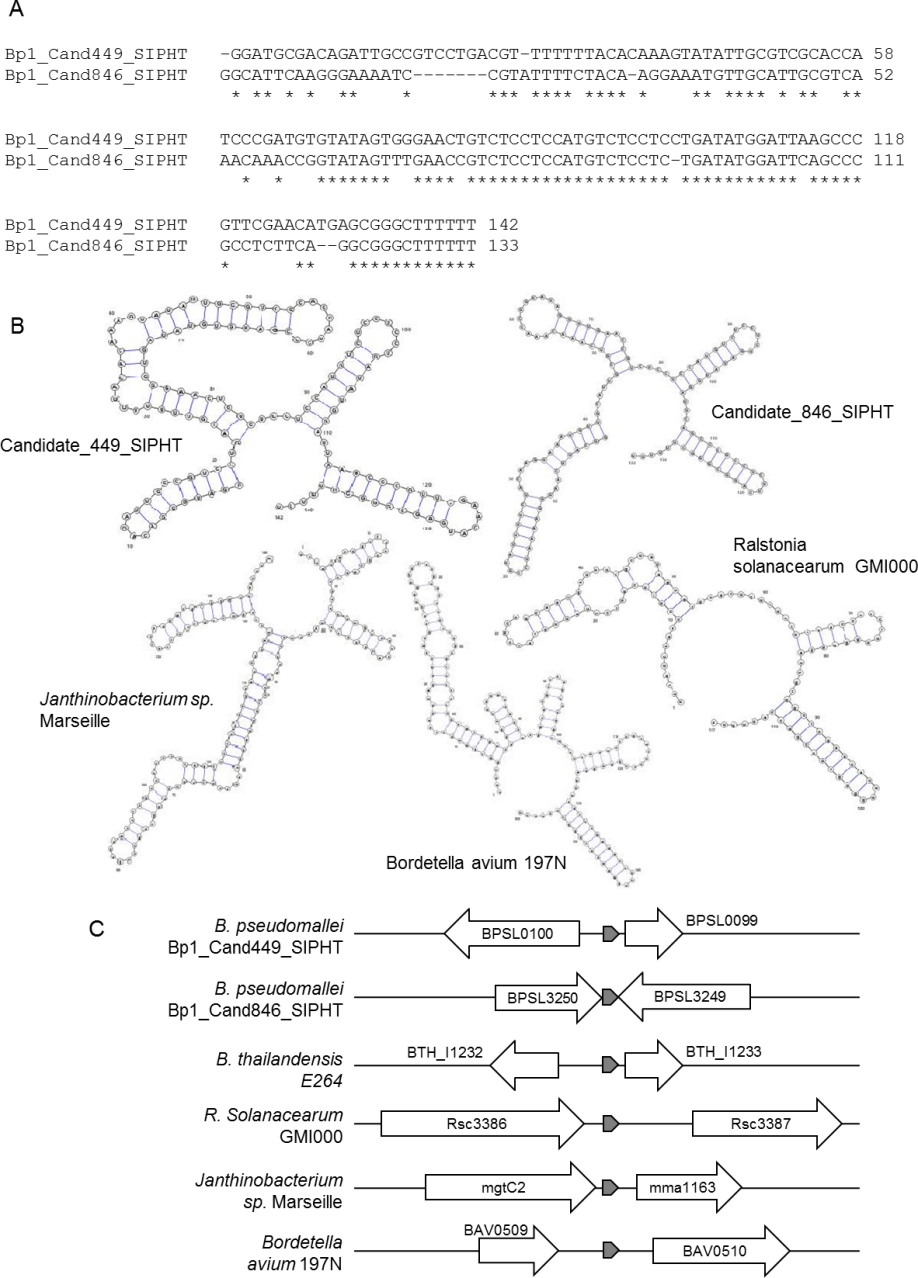
**Bp1_Cand449_SIPHT & Cand846_SIPHT**. (A) Sequence alignment of Bp1_Cand449_SIPHT and Bp1_Cand846_SIPHT. (B) Secondary structures of Bp1_Cand449_SIPHT, Bp1_Cand846_SIPHT and their homologues from *Ralstonia solanacearum, Janthinobacterium sp. Marseille *and *Bordetella avium *197N. (C) Physical co-localization for Bp1_Cand449_SIPHT and Bp1_Cand846_SIPHT and their homologues. The arrows represent the sRNA genes (shaded) and their respective flanking genes. BPSL0100 - O6-methylguanine-DNA methyltransferase, BPSL0099 - glyoxalase/bleomycin resistance protein/dioxygenase superfamily protein, BPSL3250 - putative LysR-family transcriptional regulator, BPSL3249 - putative outer membrane protein, BTH_I1232 - OsmC/Ohr family protein, BTH_I1233 - ribosomal protein L13, Rsc3386 - outermembrane signal peptide protein, Rsc3387 - Two-component response regulator transcription regulator protein, mgtC2 - Magnesium transporter accessory protein, mma1163 - Transcriptional regulator-like protein, BAV0509 - Hypothetical protein, BAV0510 - O-antigen biosynthesis glucosyltransferase.

In this section we analyse and discuss several of the novel and validated sRNA genes in our *B. pseudomallei *D286 model that are the end results of the initial gene prediction and comparative analysis using our pipeline. Bp1_Cand449_SIPHT and Bp1_Cand846_SIPHT, which are highly conserved in *Burkholderia*, have a 71.7% sequence identity and highly similar predicted secondary structures (Figure 5A,B). A consensus was found for the two putative sRNAs in the Infernal search (i.e. two distinct Infernal searches for the two sRNAs returned same locations in several genomes, including *R. solanacearum, Bordetella avium *and *Janthinobacterium sp*. (Figure 5B). From the Infernal search, homologues of this sRNA were detected in bacteria of different order, class and phylum, with one or two copies in each genome. In addition to Burkholderiales, the two sRNAs were also detected in Actinomycetales, Bacillales, Enterobacteriales, Neisseriales, Pseudomonadales as well as Vibrionales. This proposes the possibility that these two sRNA genes are paralogues of each other and not artifacts of a sequence assembly error. As the gene distribution for this putative sRNA covers a wide range of evolutionary distances (Gram positive to Gram negative) and exhibited a low evolutionary rate, it is also quite plausible that Bp1_Cand449_SIPHT and Bp1_Cand846_SIPHT are involved in essential bacterial pathways. The physical co-localization of Bp1_Cand449_SIPHT, Bp1_Cand846_SIPHT and their homologues in *B. thailandensis, R. solanacearum, Bordetella avium *and *Janthinobacterium sp*. were visualized and investigated (Figure 5C). It was found that genes located directly upstream and downstream of the sRNAs were dissimilar in different species. Moreover, the distances between these sRNAs and their flanking genes were also different in each of the genomes. This suggests that these sRNAs are either trans-acting elements or a generic type of cis regulator that can be present in different regulatory pathways.

**Figure 6:**
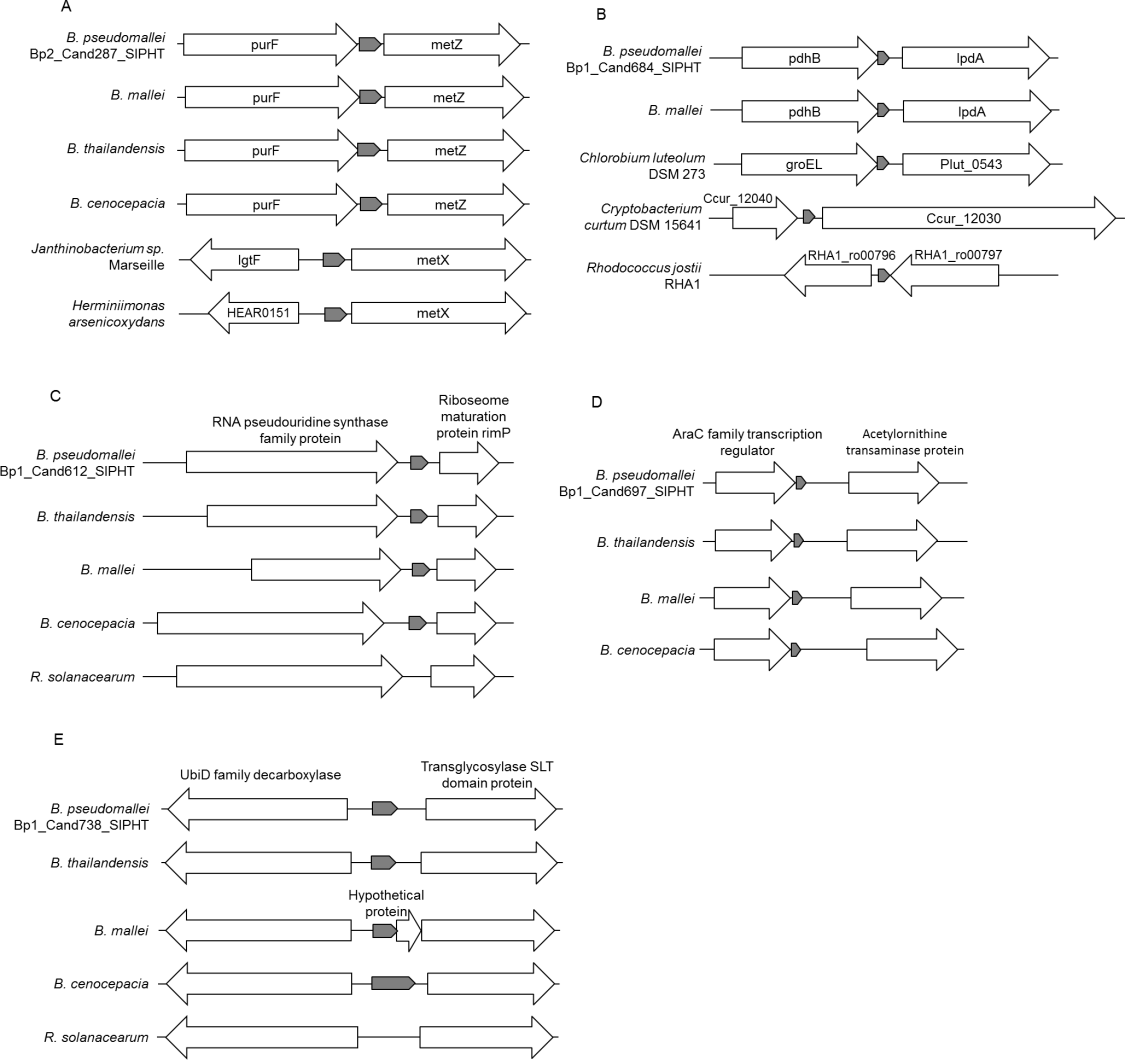
**Physical co-localization for verified sRNAs**. The arrows represent the sRNA genes (shaded) and the flanking genes. (A) Bp2_Cand287_SIPHT: *purF *codes for amidophosphoribosyltransferase; *lgtF *codes for UDP-glucose-lipooligosaccharide glucosyltransferase; *HEAR0151 *codes for chromate transporter; metZ codes for O-succinylhomoserine sulfhydrylase; metX codes for homoserine O-acetyltransferase. (B) Bp1_Cand684_SIPHT. pdhB codes for dihydrolipoamide acetyltransferase; lpdA codes for dihydrolipoamide dehydrogenase LpdA; groEL codes for molecular chaperone GroEL; Plut_0543 codes for exoenzyme S synthesis protein B; Ccur_12040 codes for ATP synthase, F1 epsilon subunit; Ccur_12030 codes for DNA-directed DNA polymerase III PolC; RHA1_ro00796 codes for hypothetical protein; RHA1_ro00797 codes for nitroreductase. (C) Bp1_Cand612_SIPHT. (D) Bp1_Cand697_SIPHT. (E) Bp1_Cand738_SIPHT

The homologues of Bp1_Cand287_SIPHT were not only detected in Burkholderiales, but also in δ-proteobacteria and γ-proteobacteria. From the physical co-localization analysis, the sRNAs were located upstream of genes that are involved in methionine metabolism in most of the genomes (Figure 6A). The sRNA was therefore suggested to be a cis-acting element involved in the modulation of methionine metabolism.

Bp1_Cand684_SIPHT was detected in different groups of bacteria, including Actinobacteria, Cyanobacteria and Firmicutes. Physical co-localization analysis showed that the flanking genes were not associated with the same pathways or functions (Figure 6E), suggesting a possible trans-acting role.

Bp1_Cand612_SIPHT, Bp1_Cand697_SIPHT and Bp1_Cand738_SIPHT are RT-PCR validated sRNA candidates that were found to be *Burkholderia*-specific. These three sRNAs were not detected in bacteria other than *Burkholderia *spp. during the Infernal search. From the physical co-localization analysis, each of these three sRNA genes has similar flanking genes in different *Burkholderia *spp. (Figure 6B-D). For Bp1_Cand612_SIPHT and Bp1_Cand697_SIPHT, although *R. solanacearum *has a similar gene arrangement at the equivalent regions, no such sRNA genes were predicted in that genome.

**Figure 7:**
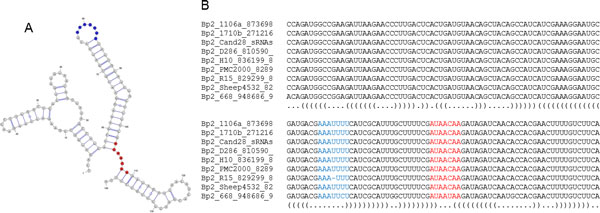
**Bp2_Cand11_sRNAscanner**. Consensus secondary structure predicted by RNAalifold (A) using the annotated alignment generated by LocARNA as shown in (B). The highlighted region in the alignment are putative Hfq-binding motifs.

In prokaryotes, Hfq proteins regulate translation by modulating the structure of numerous RNA molecules. The motif 5'-AAYAAYAA-3' is enriched in Hfq-binding RNAs and binding to Hfq was confirmed by DMS footprinting [60] while other researchers have shown that Hfq binds sRNAs with a preference for AU-rich sequences [61-63]. One of the identified *B. pseudomallei*-specific sRNAs, Bp2_Cand11_sRNAscanner, was found to contain a putative Hfq-binding motif (5'-AAYAAYAA-3') and several AU-rich regions. The secondary structures of the sRNAs showed that the motif and one of the AU-rich region were accessible (i.e. located at the loop region), implying that Bp2_Cand11_sRNAscanner could be a Hfq-dependent RNA (Figure 7).

## Conclusions

A total of 1,306 *B. pseudomallei *sRNA genes were predicted in this study of which: 21 have homologs in Rfam; 15 novel sRNAs were shortlisted due to their conservation in *Burkholderia *spp. or different *B. pseudomallei *strains; and 8 of these were verified experimentally. Though the functions for the novel sRNAs obtained in this study remain unknown, their presence in *B. pseudomallei *is evidence that sRNAs are indeed involved in this bacterium's many different cellular activities that may include regulation of pathogenesis and virulence mechanisms as well as adaptation to environmentally induced changes.

## Competing interests

The authors declare that they have no competing interests.

## Authors' contributions

KJS developed the sRNA prediction pipeline and carried out the computational sRNA discovery and comparative analyses. SN contributed to the genome sequence analysis of the *B. pseudomallei *strains. CSF carried out the validation experiments. KJS, CSF and MFR wrote the manuscript. RM contributed to the design and execution of the validation experiments. SN and MFR revised the manuscript. All authors submitted comments, read and approved the final manuscript.

## Supplementary Material

Additional file 1 **Bacterial genomes studied**. List of genome sequences used for sRNA prediction and analysis. Click here for file

Additional file 2 **Primers**. Nucleotide sequences of PCR primers for amplifying sRNA genes. Click here for file

Additional file 3 **sRNA candidates**. List of sRNA candidates predicted in fourteen bacterial genomes. Click here for file

Additional file 4 **Comparative analysis**. The results of comparative analysis of sRNA candidates predicted. Click here for file

Additional file 5 **Secondary structures of known sRNAs identified**. Secondary structures visualization for known sRNAs discovered in this study and reference structures predicted from Rfam entries. Click here for file

Additional file 6 **Infernal search results**. The results of putative sRNA homologues search using Infernal. Click here for file
