# Supplementary material for: Computational discovery and RT-PCR validation of novel Burkholderia conserved and Burkholderia pseudomallei unique sRNAs
Source: BMC Genomics. 2012 Dec 7;13(Suppl 7):S13. doi: 10.1186/1471-2164-13-S7-S13 (PMC3521395; doi:10.1186/1471-2164-13-S7-S13)
Supplement: Additional file 1 — Bacterial genomes studied. List of genome sequences used for sRNA prediction and analysis. [file 1471-2164-13-S7-S13-S1.docx]

Genome sequences used in the study. Data downloaded on 3^rd^ January 2012.

| **No.** | **Organism** | **Chr.** | **Abbr.** | **Accession** | **Size (Mb)** | **Ref** |
| --- | --- | --- | --- | --- | --- | --- |
| *sRNA gene prediction* | | | | | | |
| 1. | *Burkholderia* *pseudomallei* K96243 | 1 | Bp1 | BX571965 | 4.07 | [1] |
|  |  | 2 | Bp2 | BX571966 | 3.17 |  |
| 2. | *Burkholderia* *mallei* ATCC 23344 | 1 | Bm1 | CP000010 | 3.51 | [2] |
|  |  | 2 | Bm2 | CP000011 | 2.33 |  |
| 3. | *Burkholderia* *thailandensis* E264 | 1 | Bt1 | CP000086 | 3.81 | [3] |
|  |  | 2 | Bt2 | CP000085 | 2.91 |  |
| 4. | *Burkholderia* *glumae* BGR1 | 1 | Bg1 | CP001503 | 3.91 | (unpublished) |
|  |  | 2 | Bg2 | CP001504 | 2.83 |  |
| 5. | *Burkholderia* *phymatum* STM815 | 1 | Bpm1 | CP001043 | 3.48 | [4] |
|  |  | 2 | Bpm2 | CP001044 | 2.70 |  |
| 6. | *Burkholderia* *phytofirmans* PsJN | 1 | Bpt1 | CP001052 | 4.47 | (unpublished) |
|  |  | 2 | Bpt2 | CP001053 | 3.63 |  |
| 7. | *Burkholderia* *cenocepacia* J2315 | 1 | Bc1 | AM747720 | 3.87 | [5] |
|  |  | 2 | Bc2 | AM747721 | 3.22 |  |
|  |  | 3 | Bc3 | AM747722 | 0.88 |  |
| 8. | *Burkholderia* *ambifaria* AMMD | 1 | Ba1 | CP000440 | 3.56 | [6] |
|  |  | 2 | Ba2 | CP000441 | 2.65 |  |
|  |  | 3 | Ba3 | CP000442 | 1.28 |  |
| 9. | *Burkholderia* *multivorans* ATCC 17616 | 1 | Bmv1 | CP000868 | 3.45 | [7, 8] |
|  |  | 2 | Bmv2 | CP000869 | 2.47 |  |
|  |  | 3 | Bmv3 | CP000870 | 0.92 |  |
| 10. | *Burkholderia* *vietnamiensis* G4 | 1 | Bv1 | CP000614 | 3.65 | (unpublished) |
|  |  | 2 | Bv2 | CP000615 | 2.41 |  |
|  |  | 3 | Bv3 | CP000616 | 1.24 |  |
| 11. | *Burkholderia* *xenovorans* LB400 | 1 | Bx1 | CP000270 | 4.90 | [9] |
|  |  | 2 | Bx2 | CP000271 | 3.36 |  |
|  |  | 3 | Bx3 | CP000272 | 1.47 |  |
| 12. | *Ralstonia* *eutropha* H16 | 1 | Re1 | AM260479 | 4.05 | [10] |
|  |  | 2 | Re2 | AM260480 | 2.91 |  |
| 13. | *Ralstonia pickettii* 12J | 1 | Rp1 | CP001068 | 3.94 | (unpublished) |
|  |  | 2 | Rp2 | CP001069 | 1.30 |  |
| 14. | *Ralstonia solanacearum* GMI1000 | 1 | Rs | AL646052 | 3.72 | [11] |
| *Comparative analysis* | | | | | | |
| 1. | *Burkholderia pseudomallei* 1106a | 1 | - | NC_009076 | 3.99 | (unpublished) |
|  |  | 2 | - | NC_009078 | 3.10 |  |
| 2. | *Burkholderia pseudomallei* 1710b | 1 | - | NC_007434 | 4.13 | (unpublished) |
|  |  | 2 | - | NC_007435 | 3.18 |  |
| 3. | *Burkholderia pseudomallei* 668 | 1 | - | NC_009074 | 3.91 | (unpublished) |
|  |  | 2 | - | NC_009075 | 3.13 |  |
| 4. | *Burkholderia pseudomallei* MSHR346 | 1 | - | NC_012695 | 4.10 | (unpublished) |
| 5. | *Burkholderia pseudomallei* D286 | 1 | - | (local strain) | 3.68 | (unpublished) |
|  |  | 2 | - | (local strain) | 2.90 |  |
| 6. | *Burkholderia pseudomallei* H10 | 1 | - | (local strain) | 3.81 | (unpublished) |
|  |  | 2 | - | (local strain) | 3.00 |  |
| 7. | *Burkholderia pseudomallei* PMC2000 | 1 | - | (local strain) | 3.73 | (unpublished) |
|  |  | 2 | - | (local strain) | 2.96 |  |
| 8. | *Burkholderia pseudomallei* R15 | 1 | - | (local strain) | 3.76 | (unpublished) |
|  |  | 2 | - | (local strain) | 2.98 |  |
| 9. | *Burkholderia pseudomallei* Sheep4523 | 1 | - | (local strain) | 3.78 | (unpublished) |
|  |  | 2 | - | (local strain) | 2.99 |  |

**References:**

1. Holden MT, Titball RW, Peacock SJ, Cerdeno-Tarraga AM, Atkins T, Crossman LC, Pitt T, Churcher C, Mungall K, Bentley SD, et al: Genomic plasticity of the causative agent of melioidosis, *Burkholderia* *pseudomallei*. *Proc Natl Acad Sci U S A* 2004, 101:14240-14245.

2. Nierman WC, DeShazer D, Kim HS, Tettelin H, Nelson KE, Feldblyum T, Ulrich RL, Ronning CM, Brinkac LM, Daugherty SC, et al: Structural flexibility in the *Burkholderia mallei* genome. *Proc Natl Acad Sci U S A* 2004, 101:14246-14251.

3. Kim HS, Schell MA, Yu Y, Ulrich RL, Sarria SH, Nierman WC, DeShazer D: Bacterial genome adaptation to niches: divergence of the potential virulence genes in three *Burkholderia* species of different survival strategies. *BMC Genomics* 2005, 6:174.

4. Vandamme P, Goris J, Chen WM, de Vos P, Willems A: *Burkholderia tuberum* sp. nov. and *Burkholderia phymatum* sp. nov., nodulate the roots of tropical legumes. *Syst Appl Microbiol* 2002, 25:507-512.

5. Holden MT, Seth-Smith HM, Crossman LC, Sebaihia M, Bentley SD, Cerdeno-Tarraga AM, Thomson NR, Bason N, Quail MA, Sharp S, et al: The genome of *Burkholderia cenocepacia* J2315, an epidemic pathogen of cystic fibrosis patients. *J Bacteriol* 2009, 191:261-277.

6. Coenye T, Mahenthiralingam E, Henry D, LiPuma JJ, Laevens S, Gillis M, Speert DP, Vandamme P: *Burkholderia ambifaria* sp. nov., a novel member of the Burkholderia cepacia complex including biocontrol and cystic fibrosis-related isolates. *Int J Syst Evol Microbiol* 2001, 51:1481-1490.

7. Nagata Y, Matsuda M, Komatsu H, Imura Y, Sawada H, Ohtsubo Y, Tsuda M: Organization and localization of the *dnaA* and *dnaK* gene regions on the multichromosomal genome of *Burkholderia multivorans* ATCC 17616. *J Biosci Bioeng* 2005, 99:603-610.

8. Ohtsubo Y, Genka H, Komatsu H, Nagata Y, Tsuda M: High-temperature-induced transposition of insertion elements in *Burkholderia multivorans* ATCC 17616. *Appl Environ Microbiol* 2005, 71:1822-1828.

9. Chain PS, Denef VJ, Konstantinidis KT, Vergez LM, Agullo L, Reyes VL, Hauser L, Cordova M, Gomez L, Gonzalez M, et al: *Burkholderia xenovorans* LB400 harbors a multi-replicon, 9.73-Mbp genome shaped for versatility. *Proc Natl Acad Sci U S A* 2006, 103:15280-15287.

10. Pohlmann A, Fricke WF, Reinecke F, Kusian B, Liesegang H, Cramm R, Eitinger T, Ewering C, Potter M, Schwartz E, et al: Genome sequence of the bioplastic-producing "Knallgas" bacterium *Ralstonia eutropha* H16. *Nat Biotechnol* 2006, 24:1257-1262.

11. Salanoubat M, Genin S, Artiguenave F, Gouzy J, Mangenot S, Arlat M, Billault A, Brottier P, Camus JC, Cattolico L, et al: Genome sequence of the plant pathogen *Ralstonia solanacearum*. *Nature* 2002, 415:497-502.
