# Supplementary material for: Computational discovery and RT-PCR validation of novel Burkholderia conserved and Burkholderia pseudomallei unique sRNAs
Source: BMC Genomics. 2012 Dec 7;13(Suppl 7):S13. doi: 10.1186/1471-2164-13-S7-S13 (PMC3521395; doi:10.1186/1471-2164-13-S7-S13)
Supplement: Additional file 2 — Primers. Nucleotide sequences of PCR primers for amplifying sRNA genes. [file 1471-2164-13-S7-S13-S2.docx]

**Nucleotide sequences of PCR primers for amplifying sRNA genes**

| **Primer** | **5'-3'** | **PCR product length(bp)** |
| --- | --- | --- |
| 449/846 F | TGCGACAGATTGCCGTCCTGA | 131 |
| 449/846 R | CCGCTCATGTTCGAACGGGC |  |
| 620 F | ACGGTTATGACACCGCGAACG | 120 |
| 620 R | AAAATGCGGCCCCGCTTTCGA |  |
| 732 F | CGGCAATAGAGGTTTGTGGGGAGT | 141 |
| 732 R | AAGCGCGACCGGAGAGTCG |  |
| 871 F | CCTTGTTGGAGTCGTCGTCGGC | 90 |
| 871 R | GAGCCCGCTAAAAACCACACGC |  |
| 738 F | GTGTTGACGATCTATAAAACCCTGC | 176 |
| 738 R | AAAAACCAGCGTCGACTGGG |  |
| 612 F | ATAATCGCGGAGTCGCTGGGC | 80 |
| 612 R | GGGCGCAACGCCCATCTTGT |  |
| 287 F | GCACGTCGCCAAAAAGGCGTG | 170 |
| 287 R | CATGCGTCGGCATCAGCGGG |  |
| 506 F | CGTTGTTTCGCTAGTTTAGACGGGA | 112 |
| 506 R | AAAAGCGCCACGGAGACCGT |  |
| 507 F | CACGGCTGATTCGCCGGC | 60 |
| 507 R | AGCGCGACACCACGAGGG |  |
| 106 F | GGTCTATCAGCGTCGCCCCT | 42 |
| 106 R | AAAAGTGACCGCCGCCCC |  |
| 697 F | GCCTGTGATGTCAGTGGTCGGT | 59 |
| 697 R | AGAAAAGTGCCCCCCGCCC |  |
| 393 F | ATTCGCCGGATCCCTGCGCG | 80 |
| 393 R | AAGCGCGCCGTGTCGTCGCGCCG |  |
| 11 F | CGAAAGGAATGCGATGACGA | 100 |
| 11 R | AAAAGCGCACGGCACTGAAACG |  |
| 77 F | CGATGGGATGCAAGCGGGAT | 61 |
| 77 R | AAAAGCGCACGGCACTGAAACG |  |
| 230 F | CGGAATTTCGCCCTCGCGGA | 75 |
| 230 R | GGAAAATGCCGCCGCCCG |  |
