# Supplementary material for: Computational discovery and RT-PCR validation of novel Burkholderia conserved and Burkholderia pseudomallei unique sRNAs
Source: BMC Genomics. 2012 Dec 7;13(Suppl 7):S13. doi: 10.1186/1471-2164-13-S7-S13 (PMC3521395; doi:10.1186/1471-2164-13-S7-S13)
Supplement: Additional file 5 — Secondary structures of known sRNAs identified. Secondary structures visualization for known sRNAs discovered in this study and reference structures predicted from Rfam entries. [file 1471-2164-13-S7-S13-S5.docx]

Secondary structure of known sRNAs identified


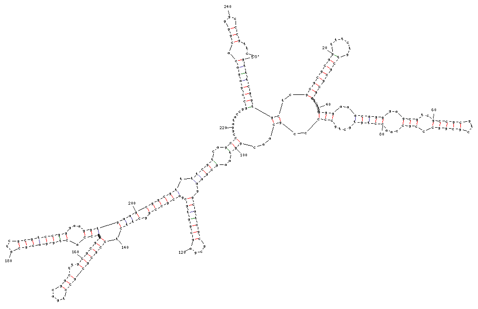

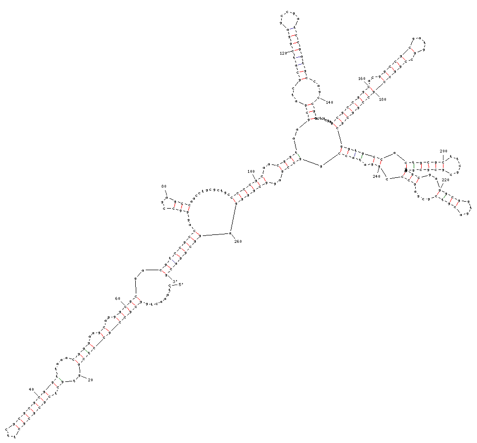

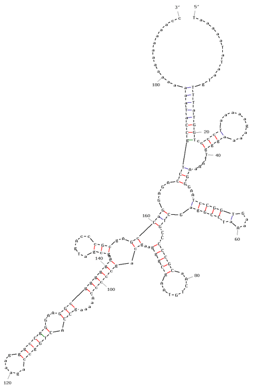


Cobalamin.1 (prediction)

Cobalamin.3 (prediction)

RF00174

(Rfam)

(A) Cobalamin riboswitch


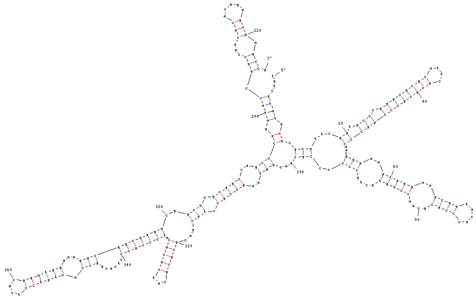


Cobalamin.4 (prediction)


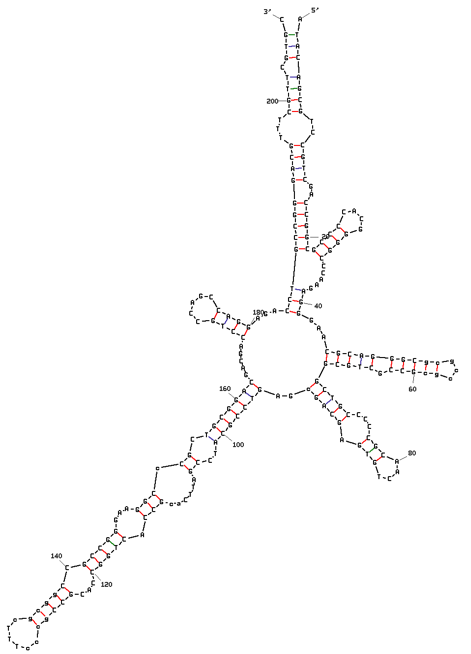


Cobalamin.2

(prediction)


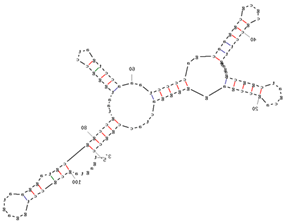

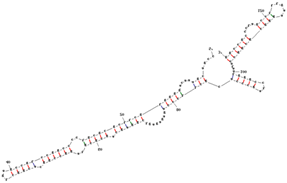

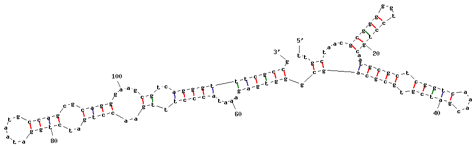


TPP.2 (prediction)

TPP.3 (prediction)


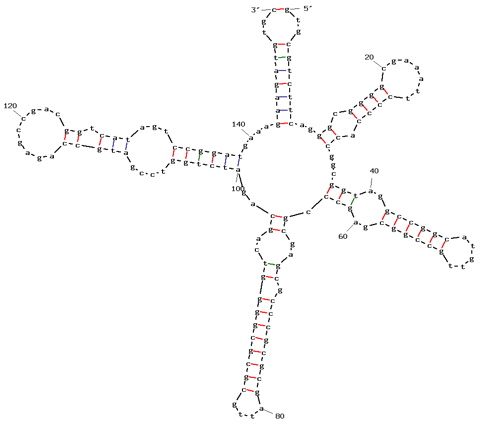

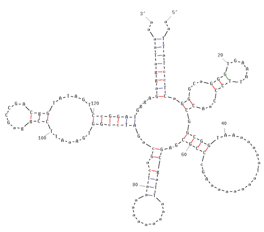


(C) FMN riboswitch / RFN element

RF00050

(Rfam)

FMN (prediction)


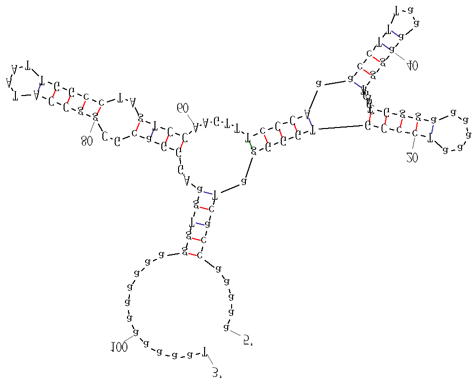


(B) TPP riboswitch

RF00059

(Rfam)

TPP.1 (prediction)


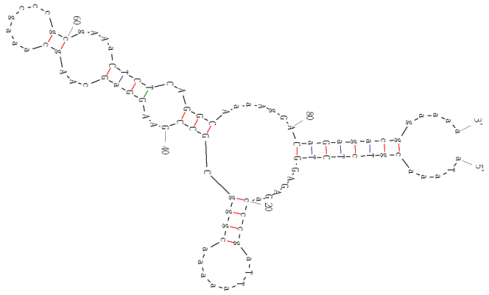

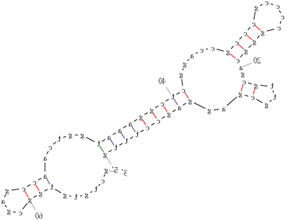

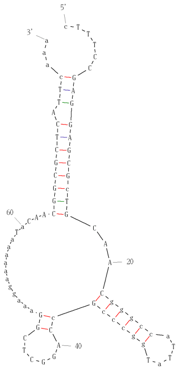


RF01057

(Rfam)

SAH_ribowitch (prediction)


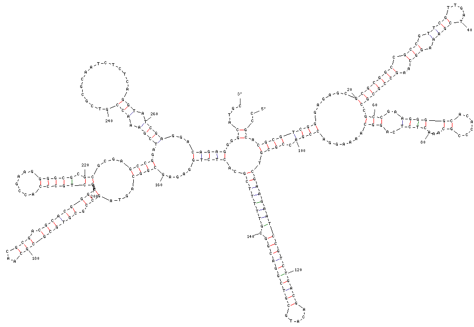


(E) Glycine riboswitch

Glycine

(prediction)


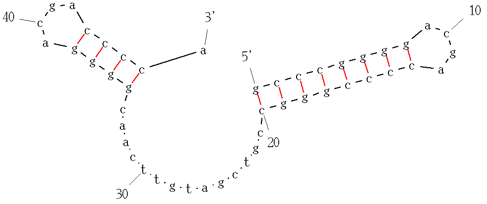

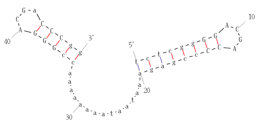


(F) Mini-ykkC RNA motif

RF01068

(Rfam)

Mini-ykkC (prediction)

(D) S-adenosyl-L-homocysteine riboswitch

RF00504

(Rfam)


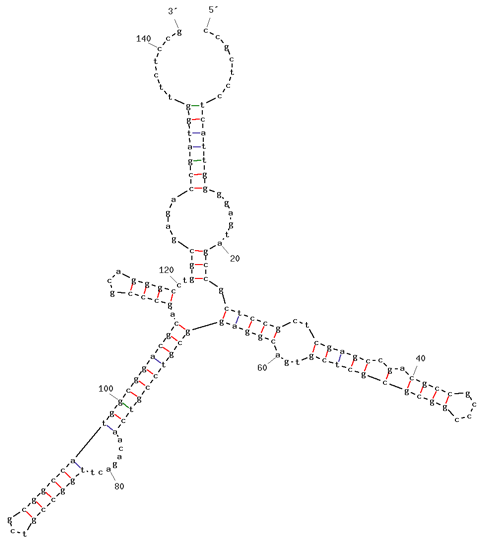

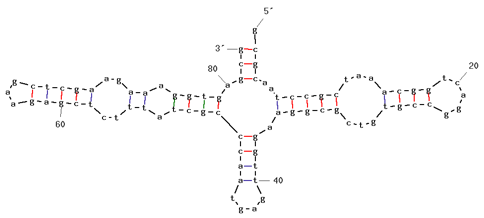

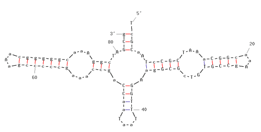

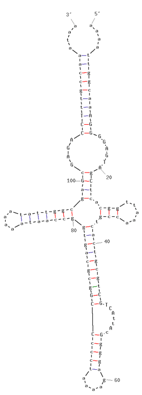


(G) sucA RNA motif


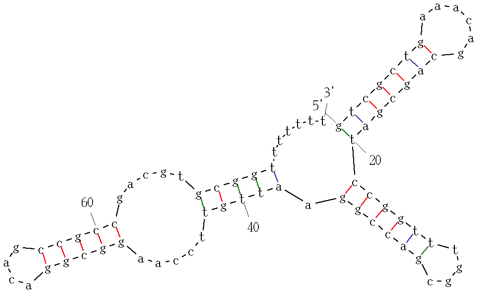

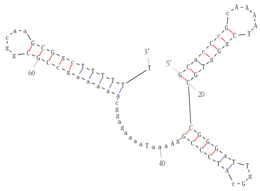


RF01070

(Rfam)

sucA (prediction)

RF00080

(Rfam)

yybP-ykoY (prediction)

RF01394

(Rfam)

isrK (prediction)

(H) yybP-ykoY leader

(I) isrK Hfq-binding RNA


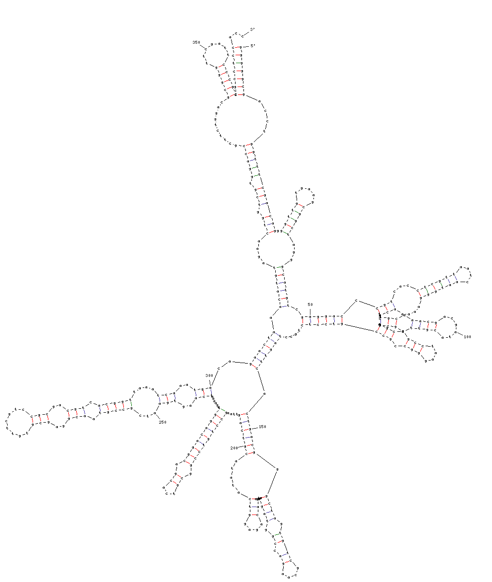

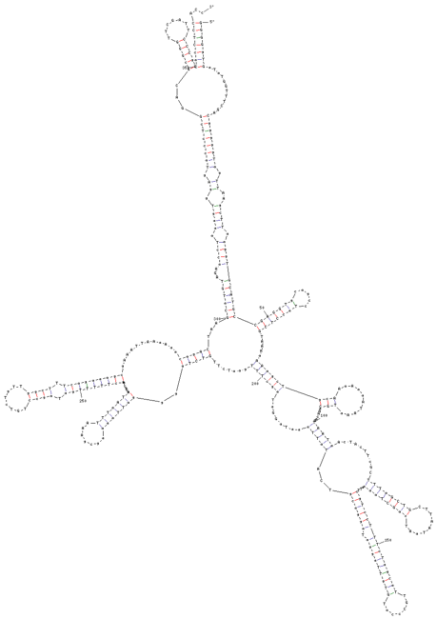

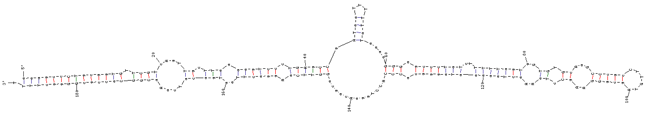

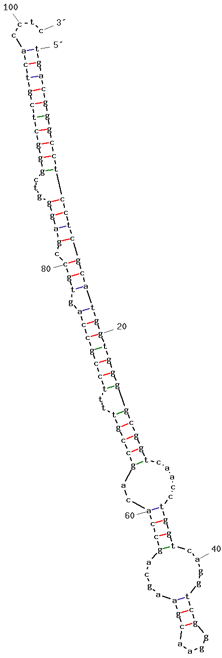

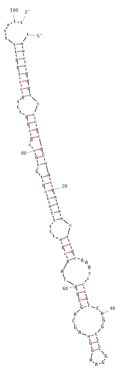


RF00169

(Rfam)

SRP_bact (prediction)

(J) Bacterial small signal recognition particle RNA


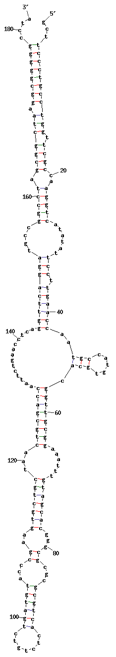


RF00013

(Rfam)

6S

(prediction)

(K) 6S RNA / SsrS

RF00023

(Rfam)

tmRNA

(prediction)

(L) Transfer messenger RNA


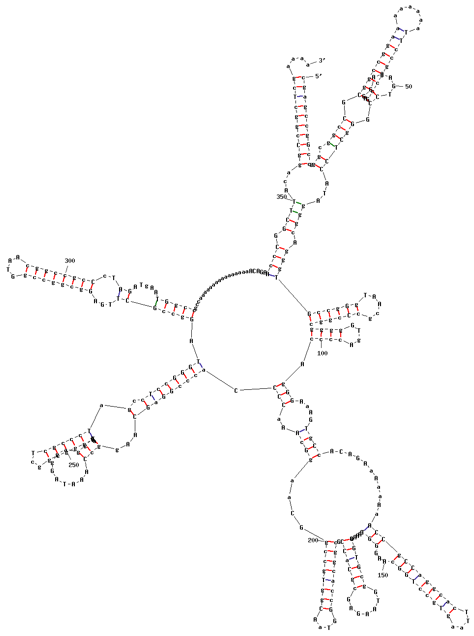

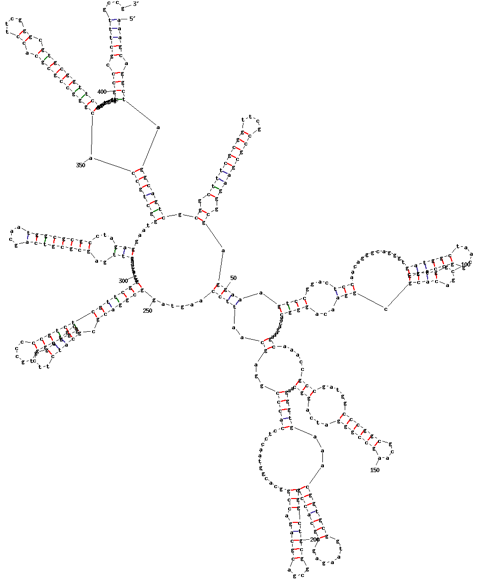

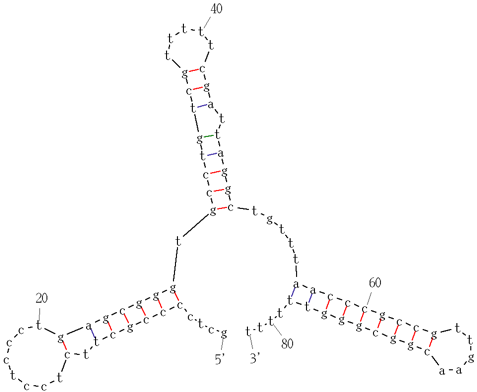


Reference:

Anti-hemB RNA motif consists primarily of two stem-loops, followed by a predicted rho-independent transcription termination stem-loop (Weinberg et al. 2007).

* Sequence and secondary structure of this RNA was not provided in Rfam

Anti-hemB

(prediction)

(N) Anti-hemB RNA motif

(M) CRISPR RNA direct repeat element


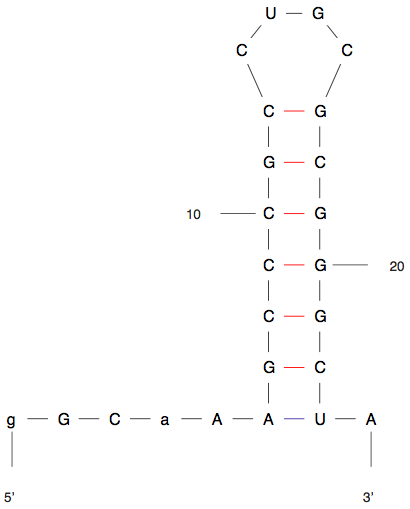


RF01356

(Rfam)


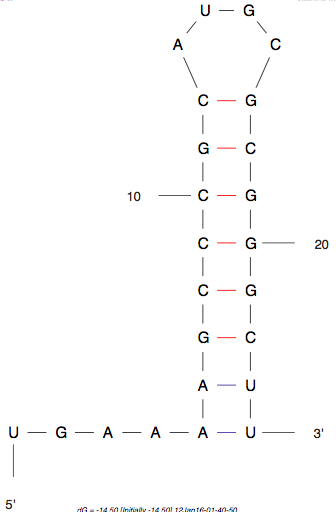


CRISPR-DR28

(prediction)

(O) Bacterial RNase P class A

RF00010

(Rfam)

RNaseP_bact_a (prediction)


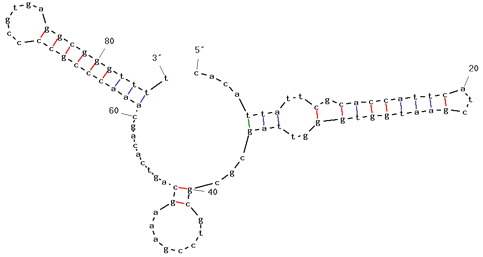

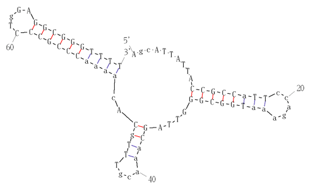


RF00624

(Rfam)

P9

(prediction)

(P) Pseudomonas sRNA P9
